# Development of a Clinical Prediction Rule for Adverse Events in Multimorbid Patients in Emergency and Hospitalisation

**DOI:** 10.3390/ijerph19148581

**Published:** 2022-07-14

**Authors:** Marta Morales-Puerto, María Ruiz-Díaz, Marta Aranda-Gallardo, José Miguel Morales-Asencio, Purificación Alcalá-Gutiérrez, José Antonio Rodríguez-Montalvo, Álvaro León-Campos, Silvia García-Mayor, José Carlos Canca-Sánchez

**Affiliations:** 1 Hospital Costa del Sol, Servicio Andaluz de Salud, Autovía A7, Km, 187 Marbella, 29603 Málaga, Spain; mamopu@hcs.es (M.M.-P.); maria.ruiz.dia.sspa@juntadeandalucia.es (M.R.-D.); purifag@hcs.es (P.A.-G.); josea.rodriguez.montalvo.sspa@juntadeandalucia.es (J.A.R.-M.); jccanca@uma.es (J.C.C.-S.); 2 Department of Nursing, Faculty of Health Sciences, Universidad de Málaga, C/Arquitecto Francisco Peñalosa 3, 29017 Málaga, Spain; jmmasen@uma.es (J.M.M.-A.); alvaroleon@uma.es (Á.L.-C.); sgmayor@uma.es (S.G.-M.); 3 Instituto de Investigación Biomédica de Málaga (IBIMA), 29590 Málaga, Spain

**Keywords:** clinical prediction rule, clinical safety, adverse events, multimorbidity, validation study, nursing

## Abstract

(1) Background: There is currently a global consensus that the quality of comprehensive care for acutely hospitalised elderly people should include addressing functionality and mobility, cognitive status, prevention of pressure ulcers, urinary incontinence, falls and delirium, as well as pain control and medication-related problems. The aim of this study is to develop and validate a clinical prediction rule for multimorbid patients admitted to an acute care hospital unit for any of the five adverse events included in our vulnerability pentad: falls, pressure ulcers, urinary incontinence, pain and delirium. (2) Methods: Longitudinal analytical clinimetric study, with two cohorts. The study population will consist of multimorbid patients hospitalised for acute care, referred from the Emergency Room. A clinical prediction rule will be proposed, incorporating predictive factors of these five adverse outcomes described. This study has received funding, awarded in November 2020 (PI-0107-2020), and was approved in October 2019 by the Research Ethics Committee ″Costa del Sol″. (3) Conclusions: Preventing adverse events in hospitalised patients is particularly important for those with multimorbidity. By applying a clinical prediction rule to detect specific risks, an estimate can be obtained of their probability of occurrence.

## 1. Introduction

It is generally agreed that the provision of comprehensive acute care for hospitalised elderly patients needs to be reviewed to avoid negative consequences of hospital stay. In this respect, many studies have recorded negative impacts produced during hospitalisation, especially among patients with multimorbidity. A cascade iatrogenesis conceptual framework describes a complex association between patients’ predisposing factors and adverse outcomes during hospitalisation. According to this framework, risk factors are synergistic among them and, consequently, removing or treating one factor in isolation is usually not sufficient to mitigate risk [1]. Thus, pursuant to these predisposing and/or precipitating factors, older people are twice as likely to suffer adverse events [2,3]. Key areas of attention for preventing such an outcome include early mobilisation, assessment of cognitive status, preventing delirium, protecting against pressure ulcers, addressing urinary incontinence, preventing falls, achieving effective control of pain and resolving medication-related issues [4].

Daily assessments of the mobility of patients hospitalised for acute care [5] have shown that length of hospital stay is reduced and physical function improved when early mobilisation is achieved [6]. With regard to falls, among hospitalised patients aged over 65 years, the number of falls suffered in the previous year is a significant predictor of functional deterioration [7]. However, the instruments currently available for assessing the risk of falls present major limitations and are not recommended for use with this population [8,9]. The prevalence of pressure ulcers in hospitals is approximately 12.8% [9]. These conditions have negative consequences on quality of life [10] and are especially dangerous for patients with multimorbidity [11]. Early assessment prevents the occurrence of pressure ulcers, but the instruments currently available are not very effective [12].

Urinary incontinence (UI) is often related to health care interventions, such as the indiscriminate use of adult diapers or bladder catheterisation. The presence of UI in previously continent elderly hospitalised patients, at the moment of discharge, has been estimated at 17%, with an odds ratio (OR) of developing UI of 4.26 (95% CI: 1.53 to 11.83) [13]. In another study [14], reported a prevalence of 26% of UI in hospitalised patients.

Among hospitalised patients, the prevalence of delirium is 23% [15], with persistent effects manifesting in 44% of patients at discharge, and still present in 21% of patients six months later, with consequent repercussions on mortality, institutionalisation, functionality, etc. [16]. Accordingly, early assessment of the risk of delirium (i.e., on admission) is of vital importance [17].

Pain is the most prevalent condition in our study population, affecting up to 23% of hospitalised multimorbid patients [18]. Its impact is greater among those with a poorer cognitive status. Moreover, 73% of these patients do not receive treatment for pain on admission and 50% do not receive it during hospital stay [19].

Another element that is strongly associated with adverse events in this population is handgrip strength, which is a predictor of mortality, length of hospital stay and use of resources. This is also correlated with walking ability, nutritional status, cognition, mobility, and functional status [20].

Another major problem associated with multimorbidity is that of polypharmacy [21], the prevalence of which has risen greatly in recent years, exceeding 20% in some areas [22] and reaching 27.3% in Spain [23]. It has been associated with various negative outcomes: delirium [24] and falls [25]. Furthermore, adverse drug reactions have been observed in 16% of these patients [26], although most studies of this subject exclude persons aged over 85 years [27]. Nevertheless, the causal direction of the association between frailty and polypharmacy remains unclear [28]. Finally, to our knowledge, no predictive models have yet been proposed to evaluate the presence of polypharmacy in hospitalised patients with multimorbidity and subject to other relevant factors [29].

As described above, the prevention of adverse events to hospitalised patients is especially important for those with multimorbidity. In this paper, we focus on five adverse events: falls, pressure ulcers, urinary incontinence, delirium and pain, termed the *pentad of vulnerability*. According to the iatrogenic cascade, these events often have synergistic effects, and early risk assessment is essential to effective intervention [1,30]. When multimorbid patients are admitted to hospital, interventions are usually focused on identifying specific risks, an approach that has been shown to be more effective than generic interventions [31]. Accordingly, it is essential to have clinical prediction rules (CPR) for this purpose, in order to estimate the probability of an event, and guiding the assessment and stratification of the hospital population considered.

Previous attempts at deriving appropriate CPR for this population have not obtained good predictive results. Many were focused on highly specific situations, and often applied lax criteria for determining the outcomes of interest [32] or employed retrospective methods in their evaluations [33]. These factors limit the value of any conclusions drawn. Moreover, a wide range of variables, indices and types of patients have been analysed [34], and the situation is further complicated by persistent conceptual weaknesses regarding vulnerability, frailty and complexity [35]. For example, the phenotypic frailty approach [36], does not produce very good results for hospitalised patients [37]. Some studies have proposed a comprehensive geriatric evaluation, but only consider doing so on admission to hospital or at certain moments during the stay [38]. Others have evaluated only the outcomes of mortality or institutionalisation, or do not incorporate predictors addressing all of the events described [39]. Another study evaluated composite tools, incorporating various risk assessment instruments. Although the authors are optimistic regarding the instruments’ predictive capacity of adverse events, only a few were included as a secondary consideration [40]. Thus, no joint evaluation was made of many of the risks known to be present (i.e., pressure ulcers, falls, urinary incontinence, delirium and pain).

As most admissions of multimorbid patients for acute hospitalisation are referred from the Emergency Room (ER), it has been suggested that this service might usefully screen vulnerable elderly patients [41], via instruments such as the Identification of Seniors at Risk (ISAR) tool, the Triage Risk Screening Tool or Variables Indicative of Placement Risk. However, the studies in question present significant limitations in terms of validity and reliability and do not distinguish between the degrees of risk facing vulnerable patients [42]. Furthermore, studies of adverse events that have taken place within the ER provide a limited representation of subjects with intrinsic risk factors, such as multimorbidity [43]. In this respect, the public health system of Andalusia (southern Spain) uses the ER Vulnerability Assessment Tool (HEVULUR, Spanish initials). This instrument measures mobility, communication, the ability to self-protect and to request help, and the existence or otherwise of a family support network [44]. However, its predictive capability regarding the incidence of adverse events in acute hospitalisation or in the ER itself has not yet been evaluated.

Multimorbid patients are especially vulnerable to dependency and severe adverse events. Therefore, the study we propose is aimed at facilitating the development of instruments to identify such risks, reliably and accurately, and at overcoming some of the knowledge gaps in this area that are currently challenging the capabilities of acute care hospitals.

### Aims

The general aim of this study is to evaluate the capabilities of various predictors of the outcome of the adverse events included in the above-described vulnerability pentad (VP) (falls, pressure ulcers, urinary incontinence, pain and delirium), in multimorbid patients.

This study has the following primary objectives: to identify predictive factors associated with the occurrence of the VP in multimorbid patients admitted to acute-care hospital units; to analyse the discriminative capacity of a rule for predicting and calibrating the compound event (PRED-VP); to determine the validity, using a delayed match-to-sample technique, of the PRED-VP prediction rule; and to assess the predictive validity of the HEVULUR instrument regarding vulnerability to adverse events in the ER (falls, delirium and/or pain).

The secondary objectives are to evaluate the association between the PRED-VP prediction rule and in-hospital and 30-day mortality; to consider whether HEVULUR accurately predicts adverse events in multimorbid patients admitted to acute-care hospital units; and to compare its performance with that of PRED-VP.

## 2. Materials and Methods

### 2.1. Design

This study will be longitudinal, analytical and clinimetric, using a validation cohort. A prospective cohort design is recommended because it enables optimal evaluation of predictors and outcomes, and the calculation of both absolute and relative risk measures [45].

### 2.2. Sample/Participants

The study population will consist of patients admitted to an acute-care hospital unit, following referral from the ER, who are considered to present multimorbidity, according to the definition given in the Comprehensive Treatment Guidelines for Multimorbid Patients, published by the Andalusian Public Health Ministry [46], i.e., presenting at least two of the following processes:

Category A: NYHA Class II heart failure, clinically stable, or ischaemic heart disease.

Category B: Vasculitis or systemic autoimmune disease. Chronic kidney disease, defined by glomerular filtration rate < 60 mL/min or albumin creatinine index > 30 mg/g.

Category C: Chronic respiratory disease, clinically stable, with MRC Class 2 dyspnoea, or FEV1 < 70%, or SaO_2_ ≤ 90%.

Category D: Inflammatory bowel disease. Chronic liver disease with evidence of hepatocellular failure (INR > 1.7, albumin < 3.5 g/dL, bilirubin > 2 mg/dL) or portal hypertension (according to clinical, laboratory, ultrasound or endoscopic data).

Category E: Cerebrovascular accident. Neurological disease with permanent motor deficit that limits the basic activities of daily life (Barthel index < 60). Neurological disease with permanent cognitive impairment.

Category F: Symptomatic peripheral artery disease. Diabetes with proliferative retinopathy or symptomatic neuropathy.

Category G: Chronic anaemia due to digestive losses or acquired haemopathy not responsive to curative treatment, presenting Hb < 10 mg/dL in two separate determinations more than three months apart. Solid or active haematologic neoplasm not responsive to curative treatment.

Category H: Chronic osteoarticular disease that limits the basic activities of daily life (Barthel index < 60). Prior osteoporotic hip fracture.

The multimorbidity criteria also include patients who meet one of the above criteria plus one or more of the following:

− Extreme polypharmacy (10 or more active prescription drugs).
− Socio-family risk (Gijón scale > 10 points).
− History of pressure ulcers classed as Stage II or higher in the past 12 months.
− Malnutrition (BMI < 18.5).
− Chronic enteral feeding.
− Two or more hospital admissions in the previous 12 months.
− Alcoholism.

Patients presenting any of the following conditions prior to hospital admission will be excluded from this study: active pressure ulcer, severe mental disorder (psychosis, bipolar disorder, schizophrenia), alteration to the urinary elimination tract requiring artificial assistive devices (bladder catheterisation, nephrostomy, urostomy), urinary incontinence, and chronic pain treated with opioids (in any form). ER patients who present acute pain evidenced by Numeric Rating Scale (NRS) > 4 maintained until hospitalisation and any patients subsequently transferred to the Intensive Care Unit (ICU) will also be excluded.

The necessary sample size will be calculated using the events per variable rule for logistic regression models [47]. The following events probabilities were estimated from the relevant literature, together with hospital information system data): falls: 2.35% [48]; pressure ulcers: 7.87% [49]; urinary incontinence: 26% [14]; delirium: 10–31% [50]; pain: 22.9%.

The VP compound variable assumes any of the above outcomes as an event. A total of 364 patients must be recruited in order to detect an event probability of 22%, with the eight predictors that will be considered. Taking into account the probability distribution of the events to be measured with the VP compound variable, this sample size is more than sufficient, as some of the outcomes greatly exceed an event probability of 22%. The feasibility of this study was assessed by consideration of the hospital’s patient recruitment capacity [47].

According to the hospital information system, in 2017, the Internal Medicine Service treated 468 patients meeting the criteria for multimorbidity. In 2018, the corresponding figure was 650, among total admissions of 2733 (17.12%) and 2788 (23.31%), respectively. Once the clinical prediction rule (CPR) has been validated, a second sample (representing the validation cohort), with the same size and subject to the same inclusion and exclusion criteria, will be analysed.

#### Materials/Instruments and Variables

The PRED-VP CPR will be designed in accordance with international standards for the development of clinical prediction rules, including the TRIPOD checklist. The Transparent Reporting of a multivariable prediction model for Individual Prognosis Or Diagnosis (TRIPOD) Initiative contains a set of recommendations for the reporting of studies developing, validating, or updating a prediction model, whether for diagnostic or prognostic purposes. The TRIPOD Statement aims to improve the transparency of the reporting of a prediction model study regardless of the study methods used. [51]. The HEVULUR instrument is already fully developed, and the proposed study will focus on determining its predictive validity, an aspect that has not been addressed since the creation of this instrument and its implementation in the Andalusian public health system.

The following predictive or independent variables will be studied: functionality: evaluated by the Barthel index [28]; history of falls: evaluated by the three screening questions recommended by the Spanish Ministry of Health [52]; risk of pressure ulcers: evaluated by the Braden scale [53]; urinary incontinence: evaluated by the ICIQ-SF [54]; pain: measured by a visual analogue scale [55,56]; delirium: evaluated by the Spanish version of the 4AT [57]; handgrip strength: evaluated by a dynamometer, on hospital admission; polypharmacy: evaluated according to the maximum number of drugs prescribed during hospital stay, assuming the commonly accepted cut-off points for polypharmacy (≥5 drugs) and hyperpolypharmacy (≥10 drugs) [23].

The primary outcome variables addressed in this study will be composite, both for acute hospitalisation and for the ER. The VP composite variable (falls, pressure ulcers, urinary incontinence, pain and delirium) for acute hospitalisation will signal the presence of any of the latter events, which will be computed as an outcome. These outcomes will also be computed separately, to estimate their individual incidence and that of the VP for adverse events within the ER (falls, pain and delirium). In every case, the occurrence of these events will be computed both as a joint outcome and independently, to determine the individual incidence.

As a secondary outcome variable, we will also study in-hospital and 30-day mortality.

The results obtained by the HEVULUR instrument on the patient’s admission to the ER and stored in the digital medical record of the hospital’s emergency department will be included in our analysis.

In addition, the following variables will be collected to adjust the analysis and to control for possible confounders or interaction factors: age, sex, reason for admission, Charlson index [58], PROFUND [59] and Pfeiffer questionnaire score [60], the latter to detect any prior cognitive impairment.

For the patients admitted, we will also record the hospital unit of admission, length of hospital stay and drugs, for special risks, prescribed during the stay: analgesics, antipsychotic medication, hypnotic-sedatives, benzodiazepines, diuretics, or drugs related to sensory-perceptual deficit prior to admission or impaired verbal communication because of clinical or language barriers.

### 2.3. Data Collection

This study will consist of the following phases:

PHASE 1: Define the CPR. Potentially predictive variables will be identified for the study population, based on a prior literature review of possible predictors, and subjected to expert judgment using the Delphi technique to generate an initial list of variables.

PHASE 2: Deduce the CPR. Multivariate logistic regression methods will be used to analyse the contribution of each variable to the predictive model. Once the model has been configured, the weights applicable to the variables will be calculated and an index of risk will be derived from the VP.

PHASE 3: Validate the HEVULUR instrument. This phase will be conducted simultaneously with Phase 2, to determine the positive and negative predictive validity of the instrument for the outcomes of fall, pain and delirium events occurring in the ER.

PHASE 4: Evaluate the secondary aims of this study. The association between the PRED-VP CPR and mortality will be determined, and the predictive capacity of the ER evaluation performed with HEVULUR will be compared with that of the PRED-VP CPR, concerning the risk of adverse events during hospitalisation.

In Phase 1, the group will include a family caregiver with experience of a family member having been admitted to an acute-care hospital unit or to the ER, together with a representative of the Malaga Chronic Disease Patients Association.

For Phases 2, 3 and 4, patients admitted to the ER will be evaluated to determine whether they meet the criteria for inclusion. If so, they will be invited to participate and their signed informed consent will be requested by the ER nurses collaborating in this study. Each patient’s eligibility for inclusion in the hospitalisation aspect of this study will be doubly verified. Once the patient has been included, the baseline data, the characterisation variables and the measurements described above will be recorded in an online encrypted database designed for this study.

**Figure 1:**
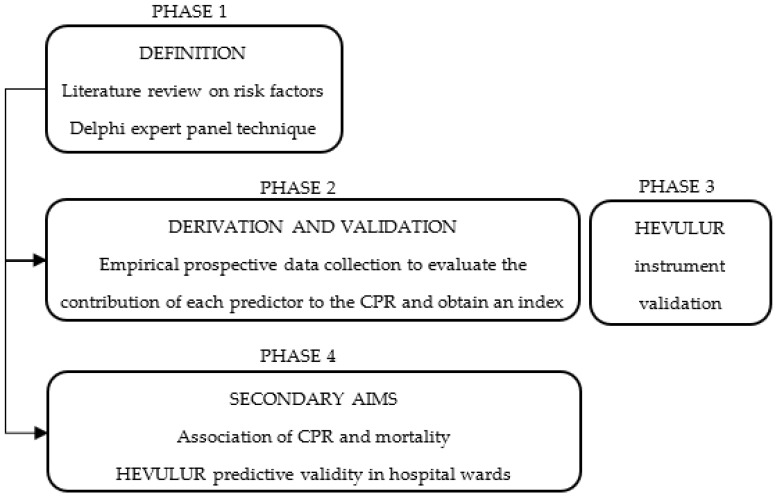
Diagram of the design and phases.

The global organisation and structure of this study are represented in Figure 1.

The evaluations will be conducted in accordance with the TRIPOD checklist, a crucial aspect of which is the independent assessment of outcomes. For this purpose, a research technician, unrelated to the health care team, will be contracted to evaluate the presence and impact of each outcome in the hospital unit and in the ER, according to the following criteria: falls (assessed both in the Emergency Room and in the hospital ward). For this purpose, the registry of falls will be reviewed daily, in addition to conducting direct interviews with patients to confirm possible undetected events. Pressure ulcers (assessed in the hospital unit). This outcome will be evaluated daily, from the patient’s clinical history and by direct examination to confirm the appearance of pressure ulcers (stage I or higher) in any area of the body. Urinary incontinence (assessed in the hospital unit). This event will be validated if any of the questions in the international consultation on incontinence questionnaire-short form (ICIQ-SF) are answered with a score > 0. Pain (assessed both in the Emergency Room, and in the hospital ward). This parameter will be evaluated daily by means of a visual analogue scale, with a threshold value of 4. Delirium (assessed both in the Emergency Room, and in the hospital ward). The presence of this condition will be assessed according to the 4AT screening criteria.

All patients will be evaluated upon admission and then every 24 h until discharge from the Emergency Room or from hospitalisation. In addition, the predictors included in the CPR will be evaluated longitudinally in the hospital unit every 48 h to determine their evolution during the patient’s stay.

### 2.4. Data Analysis

In Phase 1, the degree of agreement will be determined in accordance with the RAND corporation proposal, based on percentiles and the interquartile range. The scale used is scored from 1 to 9. Agreement is assumed to exist when at least 75% of the panellists generate median scores ranging from 1 to 3 or 7 to 9 (inclusive). Disagreement is assumed when fewer than 75% of panellists concur as above or when the median scores obtained range from 4 to 6 (inclusive) or when the interquartile range is >3. Questionnaire items that generate disagreement will be subjected to successive rounds of consideration, with feedback of the group score channelled to each panellist.

In the statistical analysis of Phases 2 to 4, in addition to the usual exploratory analysis of the variables (verifying the normality of distribution by the Kolmogorov–Smirnov test and by distribution analysis) and bivariate analysis, we will focus on the final selection of predictors, applying multivariate logistic regression to the predictors under study and performing backward stepwise regression. The fit will always include the variables age and sex, together with those incorporated in the model. The global fit of the rule will be evaluated by Nagelkerke’s R^2^ parameter, adjusted by degrees of freedom to avoid the estimation of overfitted models. Log-likelihood estimations will also be used. The calibration obtained will be evaluated using the corresponding calibration chart. The Brier score (the square of the difference between the observed events and the probability estimated by the model) will be calculated and the discrimination capacity of the rule determined using a ROC curve. Although this study is longitudinal and, a priori, Cox regression models might be considered more appropriate, logistic regression produces similar estimates in studies with a short follow-up time [61]. In our case, the follow-up time of the study cohort is short (mean hospital stay: 7.8 days) and so the latter strategy will be employed. For secondary objective No. 5 (to evaluate the association between the PRED-VP prediction rule and the in-hospital and 30-day mortality), logistic regression models will be calculated, using the procedure described above, and taking mortality as the outcome variable. For secondary objective No. 6, we will analyse the difference in event incidence by risk isogroups, according to the PRED-VP CPR, depending on the hospital admission unit considered (Internal Medicine or other service), using ANOVA or the Kruskal–Wallis test as appropriate.

### 2.5. Validity and Reliability of the Instruments Used

For each of the variables, some assessment instruments have been preselected. Table 1 shows the main validity and reliability results published for each of them.

## 3. Discussion

The predictive models that will be obtained from this study will be immediately transferrable to health service and resource management, since they are based on predictors already in clinical use and with which the professionals concerned are well acquainted. In Andalusia, the generalised use of the proposed CPR in hospitals would produce health benefits for the vulnerable, chronic and multimorbid patients who are treated daily in emergency services and hospital units, both medical and surgical.

An important aspect of this study is that it incorporates contributions from organisations of chronic patients and from family caregivers of chronic patients who have had experience of acute hospitalisation.

In terms of the immediate scope of the results obtained by this study, according to the Minimum Basic Data Set (MBDS) statistics for hospitals in Andalusia (*Estadística de Conjunto Mínimo Básico de Datos Hospitalarios*), in 2019, the Internal Medicine Services reported 109,619 hospital discharges and 1,018,165 patient-days. Assuming that 60% of hospitalised patients present multimorbidity [65], and based on the prevalence reported for the five adverse events included in the VP, which the CPR is intended to predict, the results of this study would be applicable to the detection of 1546 falls by persons in acute-care hospital units, 57,418 cases of pressure ulcer, 11,181 cases of urinary incontinence, 20,389 cases of delirium and 15,127 cases of pain. The figures are presented individually, but in real life these events often occur concurrently. In addition, our calculation only includes patients discharged from Internal Medicine Services, but in fact many patients with multimorbidity are admitted to other medical units, and therefore the number of patients who would benefit from the results of this study would be greater still.

Implementation of the PRED-VP CPR is expected to decrease the prevalence of each of the adverse events described. According to previous research in this area, programmes aimed at the early detection of delirium as a strategy to prevent falls within acute-care hospital units have achieved relative risk reductions of 45% [2,16,17,38,40,66,67]. Such a reduction, applied to the MBDS data described above and regarding only falls and delirium, would benefit over 12,000 patients. If the presence of events such as pressure ulcers, urinary incontinence or pain were reduced by 15%, some 25,000 patients would benefit. Moreover, studies have shown that, for this type of patient, interventions focused on detecting specific risks are more effective than generic actions [31].

Finally, we believe the model proposed will increase awareness among health care professionals regarding the importance of early detection of these events and may serve as a basis for future experimental studies to evaluate multicomponent interventions aimed at reducing the incidence of these adverse events and at fostering a different approach towards health care for patients at the moment of admission.

The results of this study will contribute to improving clinical safety in the care of multimorbid patients. Specifically, the predictive rule obtained will enable the stratification of risks from the moment of ER admission, enabling appropriate preventive measures to be activated, both immediately and throughout hospital stay. The rule will provide a new means of addressing and preventing adverse events and their outcomes, which currently reduce the quality of life of multimorbid patients.

This project is directly related to one of the greatest challenges currently facing our society and our health services, namely, how to deal with chronic diseases and their consequences. The framework is complex, involving chronicity, aging and increased dependency. Accordingly, research is needed to derive new approaches to the many problems that arise when health services, while highly responsive to acute processes, are inadequate in their response to increasingly common problems such as the adverse events suffered by vulnerable populations, which produce severe impacts on personal health and on the economy.

An effective clinical prediction rule based on the vulnerability pentad we describe will enable risks to be stratified from the moment of hospital admission, so that preventive measures can be taken both in the Emergency Room and throughout the patient’s hospital stay. This novel approach will contribute to preventing adverse events and outcomes, such as increased length of hospital stay, readmission and/or a worsening prognosis, all of which reduce the quality of life of vulnerable, chronic and multimorbid patients.

### Limitations

A significant limitation to this study is the possibility of sample losses and missing values, regarding predictors and/or outcomes. To minimise this potentially critical problem, a research technician will be present throughout this study, both in the ER and in the acute-care unit, providing an independent daily record of the adverse events that may occur.

In addition, the study team will be composed of health care professionals from the ER and from the Internal Medicine Service, collaborating in data collection and reinforcing the support structure. Furthermore, the problem of missing data will be addressed by the use of multiple imputation methods, based on iterative convergence.

We acknowledge that falls may be under-reported, but the research technician will ask each of the patients included in this study, directly, about any falls experienced in order to maximise the accuracy of the data obtained.

This technician will receive on-the-job training in the independent collection of study outcomes, assessing real patients and tutored by project researchers in each of the sample collection environments (ER and hospital unit).

In predictive regression models, overfitting of the model may always occur. To minimise this possibility, only the predictors considered strictly necessary will be used, and the most parsimonious model will always be applied. In addition, the adequacy of the model will be estimated from the adjusted R^2^ value, and bootstrapping techniques will be used to estimate the fit of the model in the internal validation phase [68].

The delayed validation will be carried out on a second sample in the same hospital, rather than in a different one, in order to strictly control the study conditions in the blind evaluation of outcomes. Moreover, this method is recommended for prospective longitudinal designs such as ours [69]. Accordingly, ″geographical″ external validity will not be addressed in this study. This limitation will be reflected in the publications resulting from this study, and will be addressed in the future, when the CPR is available. In addition, patients included in this study are those referred form the Emergency Room, since this is the most likely way of admission for people with multimorbidity who suffers an acute exacerbation. Consequently, the results of this study could not be generalised to multimorbid patients admitted from other settings.

The possible impact of confounding/interaction factors will be considered, thus ensuring the presence of sufficient elements for analysis and fitting of the predictive models obtained.

## 4. Conclusions

This project is directly related to one of the greatest challenges currently facing our society and our health services, namely how to deal with chronic diseases and their consequences. The framework is complex, involving chronicity, aging and increased dependency. Accordingly, research is needed to derive new approaches to the many problems that arise when health services, while highly responsive to acute processes, are inadequate in their response to increasingly common problems such as the adverse events suffered by vulnerable populations, which produce severe impacts on personal health and on the economy. An effective clinical prediction rule based on the vulnerability pentad we describe will enable risks to be stratified from the moment of hospital admission, so that preventive measures can be taken both in the Emergency Room and throughout the patient’s hospital stay. This novel approach will contribute to preventing adverse events and outcomes, such as increased length of hospital stay, readmission and/or a worsening prognosis, all of which reduce the quality of life of vulnerable, chronic and multimorbid patients.

## Figures and Tables

**Table 1:** Validity and reliability of the instruments used.

Variable	Instrument	Validity and Reliability
Level of functionality	Barthel’s index [28] Instrument aimed to evaluate performance in activities of daily living	Cronbach’s alpha > 0.70 Satisfactory index of fit and factor loading Standardised effect size and mean standardised response between 0.68 and 1.81
One year mortality forecast	Charlson’s index [59] Prognostic tool to evaluate risk of death form comorbid conditions at ten years	Discrimination power with an area under the ROC curve of 0.510 (CI: 0.446–0.575; *p* > 0.05)
Cognitive decline	Pfeiffer questionnaire [60] Instrument for the detection of possible cognitive impairment in people over 65 years of age	Interobserver reliability 0.738 Intraobserver reliability 0.925 Internal consistency 0.82 Convergent validity 0.74 Area under ROC curve 0.89 (sensitivity 85.7; specificity 79.3) Variations according to education age
Risk of falls	History of falls screening questions [48,52]	Following NICE Guidelines against the use of fall assessment instruments in hospitals, the Spanish Ministry of Health, Social Services and Equality recommends the use of three screening questions to identify the risk of falls
Pressure ulcers	Braden scale [53] Pressure ulcer risk assessment tools	Good diagnostic performance Sensitivity: 65.69% (95% CI: 64.19–75) Specificity: 79.62% (95% CI: 78.39–80.85) PPV: 19.43% NPV: 97.37% Area under ROC curve: 0.832 (95% CI: 0.807–0.849)
Delirium in ER and hospital Unit	DRS 98 [62] A 16-item clinician-rated scale with 13 severity items and 3 diagnostic items for delirium. 4-AT [57] Delirium rapid screening tool	**DRS 98** Cronbach’s alpha 0.90 Intraclass correlation coefficient 0.98 (reliability) Cut-off scores of 15.25 and 17.75 were the two best options, giving the same sensitivity (92%), but the higher cut-off point had a higher specificity (95%) **4AT** Good diagnostic performance Sensitivity 89.7% Specificity 84.1% Area under the ROC curve: 0.93
Pain	Visual analogue scale for pain [63]	**Gallagher et al. [63]** The mean clinically significant difference between consecutive pain ratings in the consecutive pain ratings in the combined ″slightly less″ or ″slightly more″ groups was 13 mm (95% CI: 10–16 mm) **Good et al. [56]** Test–retest at 15 min: 0.73 to 0.82 Convergent validity r: 0.90 Construct validity r: 0.72 Discriminant validity r: 0.65 The instrument was significantly associated with pain reduction after treatment with *p* < 0.01 to *p* < 0.001.
Urinary incontinence	International consultation on incontinence questionnaire (ICIQ) [54]. Instrument to evaluate the frequency, severity and impact on the quality of life of urinary incontinence patients	Cronbach’s alpha: 0.89 Sensitivity: 92.1% Specificity: 55.6% PPV: 88.3% NPV: 65.9%
Handgrip strength (HGS)	Adapted handgrip [20,64] Physical test aimed to measure the maximum isometric strength of the hand and forearm muscles	Systematic review highlighting the positive association between handgrip strength and the outcome variables cognition, functional status, mobility and mortality HGS and cognition: β values 0.04 to 0.23 and HR 0.61 (HGS 90th percentile versus 10th percentile) HGS and mobility: RR: 1.85 (95% CI 1.38–2.48) HGS and functional status: Pooled coefficient 1.78 (95% CI 1.28–2.48) HGS and mortality: Combined risk coefficient 1.79 (95% CI 1.26–2.55)

## Data Availability

The datasets used and/or analysed during the current study are available from the corresponding author on reasonable request.
